# An Argument-Based Validation of an Asynchronous Written Interaction Task

**DOI:** 10.3389/fpsyg.2022.889488

**Published:** 2022-06-22

**Authors:** Ting Chen

**Affiliations:** School of Foreign Languages, Shanghai Jiao Tong University, Shanghai, China

**Keywords:** argument-based validation, asynchronous written interaction, responding to forum posts, interactional competence, language assessment

## Abstract

Interactional competence has attracted increasing attention due to its significance for language users. Previous studies concerning interactional competence mainly focus on synchronous interaction tasks, while the utilization of asynchronous interaction tasks is relatively under-explored despite the importance of asynchronous interaction in real life. Taking the “Responding To Forum Posts” (RTFP) task used in the International Undergraduate English Entrance Examination (IUEEE) at Shanghai Jiao Tong University (SJTU) as an example, the study aims to validate the use of asynchronous interaction tasks in the assessment of learner’s interactional competence. 49 students’ performances on the RTFP task were collected through a prototype test of the IUEEE in 2021. The data were analyzed through content analysis, analysis of variance, and ordinal logistic regression. The results showed that the task elicited a wide range of interactional features and test-takers at different proficiency levels differed significantly in the variety of features and the amount of emotion-based interaction. The study also found significant correlations between some of the features and test takers’ overall performance on interactional competence. The study has provided validity evidence for the RTFP task in the assessment of interactional competence and thrown light on the construct of asynchronous written interactional competence.

## Introduction

Interactional competence refers to the ability to construct a shared internal context through the joint efforts of participants in interactional language activities (Kramsch, 1986). As interactional competence is a critical and fundamental skill for language users in real life, it has attracted increasing attention from researchers and practitioners in the field of language education and language assessment (Lam, 2018). Constructs of some language education and language scales have been broadened to include interactional competence (Young, 2011; Galaczi, 2014; Galaczi and Taylor, 2018). For example, China’s Standards of English Language Ability, the English proficiency scale developed by the National Education Examinations Authority, incorporates oral interaction and written interaction into the language proficiency framework.

The current assessment of interactional competence mostly involves synchronous interactions such as interviews and paired discussions (Galaczi and Taylor, 2018). However, assessing interactional competence with synchronous tasks involves many challenges, such as test authenticity of interview-like interaction tasks and score separability of peer interaction tasks. Moreover, these types of interaction tasks are generally not time- and cost-efficient for test organizers (Ducasse and Brown, 2009). Therefore, researchers are searching for other affordable and reliable ways of assessing interactional competence (e.g., Nakatsuhara et al., 2021). Using asynchronous interaction tasks could be a possible option for the assessment of interactional competence due to its unique benefits. On the one hand, with the development and popularization of the Internet, computer-mediated interaction, such as discussions on online forums and interactions using social media software, has become increasingly common. Utilizing asynchronous interaction tasks in assessment is reflective of the target language use domain, which helps to improve the assessment authenticity. On the other hand, an asynchronous interaction task is more cost-efficient for testing organizers as it requires neither a stable platform for live information transmission nor the arrangement of human examiners. Nonetheless, although research has provided evidence for the use of asynchronous interaction tasks in assisting language learners’ acquisition of interactional competence (Chun, 1994), the use of such tasks in assessment has not been thoroughly investigated. The extant research pertaining to interactional competence has focused mainly on oral interactions, whereas asynchronous written interactions have not received extensive attention (Abe and Roever, 2019; Abe, 2021). Overall, it remains to be investigated if asynchronous interaction tasks could be employed to assess learners’ interactional competence.

Against this backdrop, this study explores the “Responding to Forum Posts” (RTFP) task in the International Undergraduate English Entrance Examination (IUEEE) at Shanghai Jiao Tong University (SJTU). Adopting an argument-based validation framework, the study uses content analysis, analysis of variance (ANOVA), and ordinal logistic regression (OLR) to investigate whether the RTFP task effectively measures the interactional competence of test-takers.

## Research Background

### Asynchronous Interaction Tasks in Language Assessment

The assessment of interactional competence entails many challenges. Using synchronous interaction tasks (e.g., paired discussions, role-playing, and interviews) to assess interactional competence generally involves a complicated process to organize the test or requires a high cost, given the fact that the test organizer needs to match test-takers for paired discussions or to arrange examiners for interviews. Moreover, computer-mediated tests featuring synchronous interaction tasks impose high technical requirements on the test platform regarding network stability due to the need for real-time transmission of information *via* audio, video, or text. Therefore, asynchronous interaction tasks have been adopted by some tests to assess interactional competence. For example, the Oxford Test of English (OTE) includes an oral interaction task in which the test-takers are required to respond to a voicemail message (Inoue et al., 2021). The IUEEE employs an RTFP task in which test-takers are required to write their responses to join the discussion after reading one post and two responses on an online forum.

Asynchronous written interaction tasks have a few unique advantages. On the one hand, asynchronous written interaction is a common language activity in real life. Using this kind of task to assess text-based computer-mediated interactional competence is reflective of the language use in the target domain, which helps to improve the context validity (see Weir, 2005). On the other hand, asynchronous written interaction tasks do not require real-time interaction and therefore have low requirements on the test platforms and the test organization. Thus, this approach is a possible choice for measuring test-takers’ interactional competence when organizations are faced with technical constraints and a limited test development budget. In addition, asynchronous interaction tasks do not have to face the challenge posed by the co-construction nature of synchronous interaction to score interpretation.

Nonetheless, evidence concerning the validity of asynchronous written interaction tasks remains limited. The question of whether this type of task can effectively measure the interactional competence of test-takers remains to be answered.

### Research on Interactional Competence

Scholars have conducted extensive research on interactional competence regarding construct, task development and scoring (Galaczi and Taylor, 2018). Previous research focuses mainly on oral interaction (e.g., Gan, 2010; Nakatsuhara et al., 2021; Zhang and Jin, 2021), whereas written interaction has received less attention (Abe and Roever, 2019; Abe, 2021). In addition, scholars have mainly addressed synchronous interaction tasks, such as oral interviews and paired discussion tasks (e.g., Galaczi, 2014; Youn, 2020), while research on asynchronous written interaction tasks remains rather limited. As existing research findings on synchronous interaction may not necessarily be generalizable to asynchronous interaction, further investigation is needed to attain a more comprehensive understanding of interactional competence.

Test-takers’ performances and rater’s feedback are the two major perspectives taken by research concerning interactional competence (Borger, 2019). Research focusing on test-taker performances summarizes the interactional features exhibited by test-takers in interaction tasks (e.g., Galaczi, 2008; Youn, 2020), while research based on rater’s feedback analyzes verbal or written rating reports to explore the interactional features that are salient to raters (e.g., Ducasse and Brown, 2009; May, 2009, 2011). These two lines of research have contributed to the operationalization of the interactional competence construct in assessments and provided an analytical framework for interactional competence. Galaczi and Taylor (2018) proposed that interactional features include turn management, topic management, interactive listening, breakdown repair and non-verbal features. To capture test-takers’ interaction in role-playing tasks, Youn (2020) coded test performances with three levels of interactional features (length of interaction, engagement with interaction and sequential organization), and found that interactional features exhibit differences across task types. For example, in oral interview tasks, test-takers mainly display the ability to answer questions instead of the capacity to initiate dialogues and change topics, while role-playing tasks can be used to assess their interactional competence with respect to conveying requests or refusals (Youn, 2020). Although participants in asynchronous written interaction tasks cannot engage in the co-construction of synchronous dialogues through turn-taking, they may still exhibit certain interactional features, such as responding to each other, asking questions or making suggestions. Therefore, previous studies on synchronous interaction tasks can inform the construction of a framework for analyzing asynchronous written interaction tasks.

Content analysis and conversation analysis are the main research methods employed by previous studies pertaining to interactional competence (Galaczi, 2008, 2013; Borger, 2019). According to Galaczi (2008), a combination of qualitative analysis and statistical analysis can provide a comprehensive perspective that can be adopted by the research of interactional competence. Therefore, this study employs mixed methods (Creswell and Plano Clark, 2017), using content analysis, ANOVA and OLR to explore the interactional features exhibited by test-takers engaged in asynchronous interaction tasks and the relationship between those features and interactional competence scores.

### Argument-Based Validation

Kane (2006, 2013) proposed and refined an argument-based approach for the validation of test score interpretation and use. This framework consists of an interpretive argument and a validity argument. First, an interpretive argument is constructed to clarify the interpretation and use of test scores and to identify the validity evidence that needs to be collected. The interpretive argument connects test-taker performance to score use through scoring, generalization and extrapolation inferences. Then, various pieces of validity evidence are collected to justify each inference under the guidance of the interpretive argument. To construct the argument for each inference, Kane adopted the Toulmin (2003) model of argument, which consists of grounds, claim, warrant, assumption, backing, qualification and rebuttal.

The argument-based validation framework has been widely used by language testing practitioners and researchers, as it allows the validity arguments to be constructed in a systematic and straightforward manner. Chapelle et al. (2008) refined Kane’s argumentation framework in the context of developing the Test of English as a Foreign Language: Internet-based Test (TOEFL iBT), and expanded the framework to include domain definition, explanation, and score use inferences. Among these inferences, the explanation inference connects test-takers’ expected scores to the test construct and is the most relevant inference with respect to construct validity.

As shown in Table 1, this study adopts the argument-based validation framework (Chapelle et al., 2008; Kane, 2013) to construct a validation framework for the RTFP task.

**Table 1:** The argument-based validation framework for the RTFP task.

Claim	The RTFP task effectively measures the test-takers’ interactional competence.
Warrant	Test performances reflect the interactional competence of test-takers at different proficiency levels.
Assumptions	(1) Responses to the RTFP task show interactional features. (2) There are differences in these interactional features across different proficiency levels. (3) The interactional features have certain influences on the test-takers’ interactional competence scores.
Sources of backings	(1) Content analysis of the test-takers’ responses to the RTFP task. (2) Analysis of the differences in interactional features exhibited by test-takers at varying performance levels. (3) Investigation of the correlation between interactional features and interactional competence scores.

### Research Questions

To investigate whether the RTFP task effectively measures interactional competence, this study proposes three research questions to test the three hypotheses raised in the aforementioned validation framework. The research questions are as follows:

1. To what extent can the RTFP task elicit interactional features?
2. Which interactional features distinguish different performance levels among test-takers?
3. How do these interactional features impact test-takers’ interactional competence scores?

## Materials and Methods

### Research Instruments

#### The RTFP Task

**Figure 1:**
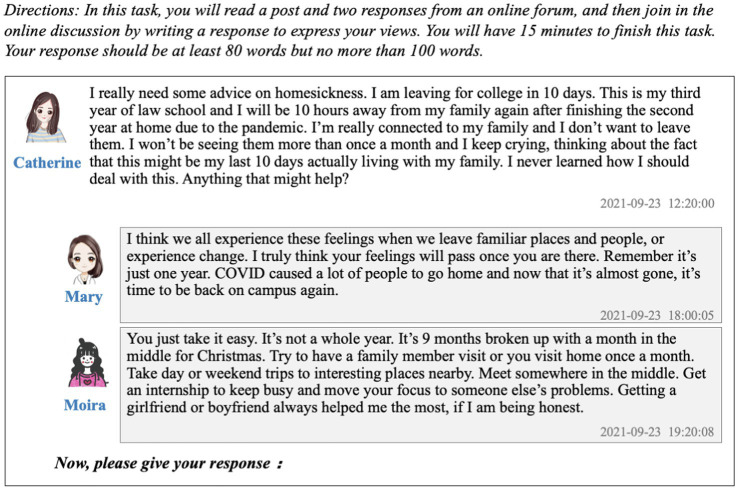
The prompts for the RTFP task.

The IUEEE is a computer-based test designed to assess whether international applicants have the necessary English proficiency to engage in undergraduate study at Chinese universities. International applicants who want to study at SJTU need to take this test, and the test scores are used to make admission decisions. The test assesses students’ ability to perform three types of language activities in an academic context: receptive activities, productive activities and interactive activities. Interactive language activities include both written interaction and oral interaction. The IUEEE employs the RTFP task to assess test-takers’ interactional competence in written interactions. In this task, test-takers are required to read one post and two responses from an online discussion forum and then to write a response between 80 and 100 words as a way of joining the discussion (see Figure 1).

#### Coding Scheme for Interactional Features

The scheme for coding interactional features was developed both deductively and inductively. The initial coding scheme was informed by previous research on interactional competence (Youn, 2020; Nakatsuhara et al., 2021) and by the inductive analysis of five test-takers’ responses. In the second stage, two coders, both of whom are PhD candidates in the field of language testing, jointly completed the coding of one test-taker’s performance and revised the coding scheme accordingly. In the third stage, the two coders each coded 5 responses. The percentage agreement was calculated to measure the inter-coder consistency. At this stage, the coding consistency was 85.11%. Coding disagreements were discussed, and the coding scheme was revised again. In the fourth stage, the two coders coded the remaining responses independently. Finally, coding discrepancies were discussed until a consensus was achieved. The inter-coder agreement rate was 81.60% at this stage. The final coding scheme is shown in Table 2.

**Table 2:** The coding scheme for interactional features.

Codes	Examples from the test-takers’ responses
A. Displaying understanding	
A1 Displaying understanding of the original post	
A1.1 Directly expressing an understanding of the post writer’s feelings	I understand your feelings.
A1.2 Analyzing and evaluating the situation and problems faced by the post writer	Also 10 h away from your family is not that distanced as of those students living in different country with different time of zone.
A1.3 Sharing similar experiences of one’s own or others	Because of the pandemic, I have not been back to my hometown for almost 2 years.
A2 Displaying understanding of the replies	
A2.1 Expressing agreement or disagreement with the opinions expressed in the replies	I agree with Moira regarding getting an internship.
A2.2 Explaining one’s reasons for agreeing or disagreeing with the opinions expressed in the replies	Because leaving your family means when you are completely on your own you should utilize this precious time to improve yourself in various ways.
B Projecting actions	
B1 Making suggestions	Try to have some social activities during your other times at school.
B2 Providing reasons to support one’s suggestions	I think it would help you may pay less concentration to the feelings on your family.
B3 Expressing comfort or encouragement to the post writer	May we all become happier in this pandemic.

The final coding scheme classified the interactional features uncovered in test-takers’ performances into two broad categories: (A) displaying understanding and (B) projecting actions. More specifically, displaying understanding was divided into (A1) displaying understanding of the original post and (A2) displaying understanding of the replies. Displaying understanding of the original post was further divided into three subcategories: (A1.1) directly expressing an understanding of the post writer’s feelings, (A1.2) analyzing and evaluating the situation and problems faced by the post writer, and (A1.3) sharing similar experiences by oneself or others. Displaying understanding of the replies was divided into two subcategories: (A2.1) expressing agreement or disagreement with the opinions expressed in the replies and (A2.2) explaining one’s reasons for agreeing or disagreeing with the opinions. The category of projecting actions was divided into three subcategories: (B1) making suggestions, (B2) providing reasons to support one’s suggestions, and (B3) expressing comfort or encouragement to the post writer. In total, the final coding scheme included eight types of interactional features.

The T-unit was used as the unit of analysis in the present study. The concept of the T-unit was proposed by Hunt (1965). It refers to the minimal terminable unit, which consists of a main clause and its subordinate clauses. The T-unit has been commonly used to conduct content analysis in linguistic research. A total of 49 responses were coded into 432 T-units, with an average of 8.82 T-units for each test-taker’s response.

### Participants

The data were drawn from the prototype test of the IUEEE in 2021. A total of 49 undergraduate international students studying at SJTU participated in the test. The proportions of test-takers who reported pursuing majors in natural sciences and engineering, humanities and arts, and social sciences were 34.69, 30.61, 34.69%, respectively. Students from non-Asian countries and students from Asian countries accounted for 55.10, 44.90% of the total, respectively. Males and females accounted for 61.22, 38.78%, respectively.

### Data Collection

The data collection was divided into two stages. During the first stage, participants were recruited to take the test, and test performance data were collected. The second stage involved scoring. Two raters were invited to rate the test-takers’ interactional competence on a scale of 0 to 5 according to their performance on the RTFP task. Both raters are researchers in the field of language assessment and have rich experience in English language teaching and assessment practices. After a training session, the two raters scored five performances independently. Then, the scores assigned by the two raters were compared and discussed until a consensus was reached. Finally, each rater finished the rating of the remaining 44 performances. Out of all 49 test performances, 49.98% of the interactional competence scores assigned by the two raters were exactly the same. For 95.92% of the test performances, differences in the scores assigned by the two raters were less than or equal to 1 point. Overall, the inter-rater agreement was good. The mean of the scores assigned by the two raters was used as the final score for each test-taker. Two performances that exhibited a score difference greater than 1 received a final score after discussion between the two raters. The average score of the test-takers’ interactional competence was 3.95, and the standard deviation was 0.70.

### Data Analysis

The data used in this study were the test-takers’ interactional competence scores on the RTFP task and the test-takers’ responses. The data analysis conducted for this study was divided into three stages. First, the pattern of various interactional features in the test-takers’ responses was explored through content analysis. The responses of 49 test-takers were coded for interactional features according to the coding scheme, and then the frequency and proportion of each interactional feature were calculated and analyzed. The variety of interactional features used by each test-taker was also calculated. In the second stage, ANOVA was used to determine whether test-takers with varying levels of proficiency exhibited significant differences. Finally, OLR was used to examine the relationship between the interactional features uncovered in test-takers’ performance and the test-takers’ interactional competence scores. SPSS 26.0 was used as the tool for statistical analysis in the present study.

## Results

### Analysis of Interactional Features in Test-Takers’ Responses

Table 3 presents the distribution of interactional features elicited by the RTFP task. Projecting actions was the main type of interactional feature, accounting for 64.58%, whereas displaying understanding accounted for the remaining 35.42%. Regarding the interactional features included in the displaying understanding category, displaying understanding of the original post accounted for 33.1% of the total interactional features coded, and displaying understanding of the replies accounted for only 2.31%.

**Table 3:** Frequency counts and percentages of each interactional feature.

Interactional features	Number of T-units	Percentage
A1.1 Directly expressing an understanding of the post writer’s feelings	24	5.56%
A1.2 Analyzing and evaluating the situation and problems faced by the post writer	75	17.36%
A1.3 Sharing similar experiences of one’s own or others	44	10.19%
In total (A1)	143	33.10%
A2.1 Expressing agreement or disagreement with the opinions expressed in the replies	7	1.62%
A2.2 Explaining one’s reasons for agreeing or disagreeing with the opinions expressed in the replies	3	0.69%
In total (A2)	10	2.31%
In total (A)	153	35.42%
B1 Making suggestions	147	34.03%
B2 Providing reasons to support one’s suggestions	72	16.67%
B3 Expressing comfort or encouragement to the post writer	60	13.89%
In total (B)	279	64.58%
In total	432	100.00%

Out of the eight interactional features examined, making suggestions was the most frequently observed interactional feature, accounting for 34.03% of total occurrences, followed by analyzing and evaluating the situation and problems faced by the post writer (17.36%) and providing reasons to support one’s suggestions (16.67%). Displaying an understanding of the replies was rarely observed, accounting for only 2.31%. Directly expressing an understanding of the post writer’s feeling was also observed infrequently, accounting for only 5.56% of the total.

In this study, the variety of interactional features of a test-taker is defined as the number of types of interactional features that appeared in the test-taker’s response. Regarding the variety of interactional features, each test-taker employed 3.53 types of interactional features on average. The test-takers’ performances involved at least one type and at most six types of interactional features.

The researcher proposes that the interactional features can also be divided into two categories based on the type of interaction shown by the test-takers: interaction based on content and interaction based on emotion. In emotion-based interaction, a test-taker interacts with the post writer by responding to the post writer’s emotions, such as directly expressing an understanding of the post writer’s feelings (A1.1) and expressing comfort or encouragement to the post writer (B3). Other interactional features are based on the content of the post and are therefore defined as content-based interaction. As shown in Table 4, the interactional features of test-takers were based mainly on the content, which accounted for 80.56%. Whereas the emotion-based interaction accounted for only 19.44%.

**Table 4:** Frequency counts and percentages of emotion-based interaction and content-based interaction.

	Frequency counts (T-unit)	Percentage
Emotion-based interaction	84	19.44%
Content-based interaction	348	80.56%
In total	432	100.00%

### Differences in the Interactional Features Among Test-Takers With Varying Levels of Proficiency

The test-takers were divided into a high-scoring group (top 33%, average score 4.66), a middle-scoring group (middle 33%, average score 4.00), and a low-scoring group (bottom 34%, average score 3.24) according to the ranking of their interactional competence scores. ANOVA was conducted on the interactional features exhibited by the three groups of test-takers, and the results are shown in Table 5. There was no significant difference in the number of T-units among the three groups of test-takers, but there was a significant difference in the levels of variety of interactional features they displayed. Test-takers in the high-scoring group had the highest variety of interactional features, followed by test-takers in the middle group, with those in the low-scoring group exhibiting the lowest variety of interactional features.

**Table 5:** ANOVA of interactional features among the three groups.

	Low-scoring group (N = 17)		Middle-scoring group (N = 16)		High-scoring group (N = 16)		In total (N = 49)		F	P
	mean	SD	mean	SD	mean	SD	mean	SD		
Number of T-units	8.18	2.43	9.19	1.72	9.13	1.59	8.82	1.98	1.39	0.26
Variety of interactional features	2.88	1.11	3.38	0.96	4.38	0.89	3.53	1.16	9.62^***^	0.00
Amount of emotion-based interaction	1.35	1.54	1.25	1.18	2.56	1.97	1.71	1.67	3.38^**^	0.04
Amount of content-based interaction	6.82	2.27	7.94	2.02	6.56	1.55	7.10	2.02	2.20	0.12
Percentage of emotion-based interaction (%)	15.07	16.98	13.88	13.30	26.46	17.93	18.40	16.86	2.96^*^	0.06

* Significant at the 0.1 level;

** significant at the 0.05 level; and

*** significant at the 0.01 level.

In terms of interaction types, there were significant differences among the three groups in terms of the amount and proportion of emotion-based interaction they displayed. Compared with the test-takers in the middle- and low-scoring groups, the high-scoring group exhibited the highest frequency and highest proportion of emotion-based interaction. However, there was no significant difference among the groups in terms of the amount of content-based interaction.

**Figure 2:**
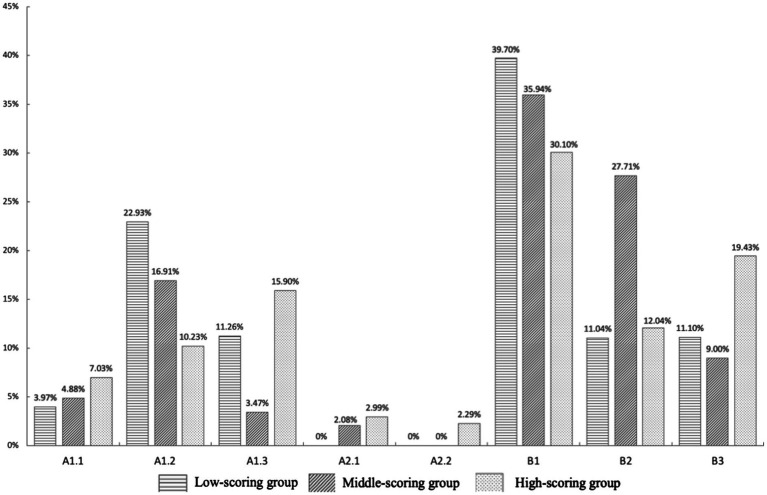
Proportion of each interactional feature in the high-scoring group, middle-scoring group, and low-scoring group.

Figure 2 shows the proportions of the eight interaction features in the performance of each group of test-takers. For test-takers at all levels, making suggestions (B1) was the most frequent interactional feature at more than 30% of occurrences and exhibited no significant differences among the three groups. Generally, compared with the test-takers in the middle- and low-scoring groups, the high-scoring group exhibited higher proportions of directly expressing an understanding of the feelings of the post writer (A1.1), sharing similar experiences (A1.3), agreeing or disagreeing with others’ replies (A2.1), explaining their reasons (A2.2) and encouraging others (B3). Test-takers in the middle- and low-scoring groups were more likely to analyze problems and make suggestions than those in the high-scoring group. In addition, only the test-takers in the middle- and high-scoring groups went beyond responding to the original post in the interaction by mentioning the replies of others in the prompts, while the test-takers in the low-scoring group did not respond to others’ replies. Overall, all types of interactional features appeared in the performance of test-takers in the high-scoring group, and the average proportion of the eight interactional features was more balanced among test-takers in the high-scoring group than among those in the middle- and low-scoring groups.

ANOVA was conducted with respect to the proportions of the eight interactional features in the performance of test-takers at varying levels. The results showed that there were significant differences among the three groups in the proportions of expressing encouragement to others (B3) and explaining the reasons for their suggestions (B2), at significance levels of 10 and 5%, respectively. Although there were differences in the proportions of other interactional features, these differences were not statistically significant. ANOVA was also conducted with respect to the quantity and proportion of displaying understanding and projecting actions among the three groups, and the results showed no significant differences.

### Correlation Between Interactional Features and Test-Takers’ Interactional Competence Scores

Based on the results reported in Section 4.2, the study further employed OLR to examine the relationship between variety of interactional features, emotion-based interaction, content-based interaction and the test-takers’ interactional competence scores. The model passed the parallelism test. The model fitness was good, and the Nagelkerke’s pseudo R-square was 0.32. The regression results are presented in Table 6. Variety of interactional features had a significant impact on the test-takers’ interactional competence scores. The more types of the interactional features were included in a test-taker’s response, the higher that test-taker’s interactional competence score. Again, the OLR results supported the existence of a positive correlation between variety of interactional features and the interactional competence score. The amount of emotion-based interaction and content-based interaction had no significant effect on test-takers’ scores.

**Table 6:** Results of OLR analysis.

	β	SE	P
Threshold (score = low)	3.75^**^	1.84	0.04
Threshold (score = middle)	5.51^***^	1.93	0.00
Variety of features	1.04^***^	0.34	0.00
Content-based interaction	0.11	0.18	0.54
Emotion-based interaction	0.10	0.22	0.65
Nagelkerke pseudo R-square	0.32		
Cox and Snell pseudo R-square	0.29		
Chi-square (model fitness)	69.70		
Chi-square (test of parallel lines)	4.38		
N	49		

** Significant at the 0.05 level; and

*** significant at the 0.01 level.

## Discussion

### Interactional Features Elicited by Asynchronous Written Interaction Tasks

The analysis revealed that the RTFP task elicited a range of interactional features from all levels of the test-takers. Projecting actions was the predominant type of interactional features across the three proficiency levels, whereas displaying understanding was less frequently observed. The three most frequent interactional features were making suggestions, providing reasons to support one’s suggestions, and analyzing and evaluating the situation and problems faced by the post writer. Regarding interaction type, the test-takers interacted mainly by providing feedback on the content of the posts and engaged in relatively less emotion-based interaction with the post writer.

This study identifies the interactional features uncovered in test-takers’ responses to asynchronous interaction tasks, which exhibit both similarities and significant differences compared to those elicited by synchronous interaction tasks. In asynchronous interaction tasks, test-takers engaged in interaction by expressing their understanding of the poster’s feelings, analyzing the situation and problems faced by the post writer, sharing similar experiences, responding to other people’s comments, making suggestions, and expressing encouragement. Among these interactional features, features such as displaying understanding of others and making suggestions are similar to those that are commonly observed in synchronous interaction tasks (Galaczi, 2008; May, 2011; Lam, 2018; Youn, 2020). On the other hand, due to the asynchronous nature of the interaction prompted by the RTFP task, the test-takers’ performance did not exhibit synchronous interactional features, such as turn management, asking for opinions, clarification, and breakdown repair.

Test-takers at different proficiency levels showed significant differences in terms of the interactional features. These differences were reflected mainly in the variety of interactional features and the amount of emotion-based interaction they displayed. The test-takers in the high-scoring group displayed a wider range of interactional features, which is consistent with previous research results concerning test-takers’ performances in oral interaction tasks (e.g., Nakatsuhara et al., 2021). In addition, the test-takers in the high-scoring group not only provided feedback to the post writer based on the content of the post but also provided more feedback pertaining to the emotions of the post writer, such as expressing an understanding of the post writer’s feelings or expressing encouragement.

As for the OLR analysis, the results showed that the variety of interactional features was positively correlated with the test-takers’ interactional competence scores, while the number of emotion-based interactions and content-based interactions had no significant impact on the scores. The reason for this result is probably that the amounts of emotion-based interactions and content-based interactions are not the key factors that determine the interactional competence scores. Instead, the relevance and effectiveness of the interaction, as well as the language used in the interaction, may affect interaction quality and therefore the interactional competence scores. As Lam (2018) argues, the scoring of interactional competence is not a simple issue; a high frequency of responses does not necessarily indicate higher interactional competence, since the quality of the response is equally important. Therefore, further research is necessary to investigate other factors that may affect test-takers’ interactional competence scores.

### The Validity Argument for the RTFP Task

The argument-based validation framework provided clear and systematic guidance for the present study. As shown in Table 7, this study combined quantitative and qualitative analysis to analyze test-takers’ performances on the RTFP task and provided support for the three hypotheses proposed under the claim that the RTFP task is able to measure test-takers’ interactional competence effectively. The study provides evidence to support the use of asynchronous written interaction tasks to examine interactional competence in language assessment practices.

**Table 7:** An argument-based validation for the RTFP task.

Claim	The RTFP task effectively measures the test-takers’ interactional competence		
Warrant	Test performances reflect the interactional competence of test-takers at different proficiency levels		
Assumptions	(1) Responses to the RTFP task show interactional features	(2) There are differences in these interactional features across different proficiency levels	(3) The interactional features have certain influences on the test-takers’ interactional competence scores.
Backings	(1) The content analysis shows that the test-takers display rich interactional features in their responses, such as displaying understanding of others, making suggestions, and providing feedback based on the content of the post and the emotions of the post writer.	(2) ANOVA shows that there are significant differences in variety of interactional features and the proportion of different types of interaction across groups at different levels.	(3) OLR analysis shows that there is a significant correlation between the variety of interactional features and the interaction competence scores of the test-takers.

## Conclusion

Taking the RTFP task in the IUEEE as an example, this study uses an argument-based validation framework to collect evidence concerning the validity of asynchronous written interaction tasks. The results of the research indicate that the RTFP task elicits a wide range of interactional features from test-takers, and there are significant differences in the variety of interactional features exhibited by test-takers at different proficiency levels. Since the ability to engage in computer-mediated written interaction has become increasingly popular and important for language learners as a result of the development of Internet technology, using computer-mediated asynchronous written interaction tasks to measure test-takers’ interactional competence is reflective of the target language use domain. In addition, the asynchronous interaction task has low technical requirements for the test organization and test platform, which improves the test practicality and operationalizability. Overall, the study argues that the asynchronous written interaction task could be utilized for examining interactional competence, especially under budget and technical constraints.

The limitations of the present study must be acknowledged. First of all, the sample size of the study is relatively small. The analysis results should be verified by examining more samples before they can be generalized. Second, this study mainly analyzes test-takers’ performance on written interaction tasks in terms of the frequency, proportion, and variety of interactional features. Other ways of portraying the interaction involved in test-takers’ performances on the RTFP task, such as strategies used in the interaction and effectiveness of the interaction, remain to be explored. For instance, it would be interesting to compare the strategy used by test-takers in asynchronous interactions to those in synchronous interactions. Third, this study adopts an argument-based validation approach and focuses on the explanation inference. Other inferences of the argument-based validation framework such as the evaluation, generalization, and extrapolation inferences need to be investigated to construct a complete validity argument. The evaluation inference, for example, needs to be justified to support the claim that the scoring of the RTFP task is appropriate. In addition, this study focuses on analyzing test-takers’ performances. Other perspectives may be adopted to reveal new implications on the nature of interactional competence. For example, rater orientation studies can be conducted in the future to further enrich our understanding of the construct of interactional competence in asynchronous interactions. Despite the limitations, this study has contributed some insight into the assessment of interactional competence. It is a preliminary exploration of the asynchronous written interaction task and shows the possibility to broaden the construct that can be elicited by an asynchronous interaction task.

## Data Availability Statement

The raw data supporting the conclusions of this article will be made available by the authors, without undue reservation.

## Author Contributions

TC contributed to the conceptualization, the literature research, the data analysis, the writing of the manuscript, and the design of the tables and figures.

## Funding

This work was supported by a grant from the International Affairs Division of Shanghai Jiao Tong University (Grant number: WF610560516/001).

## Conflict of Interest

The author declares that the research was conducted in the absence of any commercial or financial relationships that could be construed as a potential conflict of interest.

## Publisher’s Note

All claims expressed in this article are solely those of the authors and do not necessarily represent those of their affiliated organizations, or those of the publisher, the editors and the reviewers. Any product that may be evaluated in this article, or claim that may be made by its manufacturer, is not guaranteed or endorsed by the publisher.
